# Towards an Evolutionary View of Bipolar Disorders

**DOI:** 10.2174/1745017901814010233

**Published:** 2018-09-29

**Authors:** Antonio E. Nardi

**Affiliations:** Institute of Psychiatry – Federal University of Rio de Janeiro. National Academy of Medicine, Rio de Janeiro, Brazil

During the last decades, there has been a strong emphasis on the concern how environmental features, for example nutritional or toxicological conditions, can influence brain metabolism and biochemistry and human behaviour [1, 2]. We all recognize that the environment may impact our health, a special attention has been given to mechanisms related to mental health and mental disorder [1]. Nowadays, it is clear that environmental exposures and neuroendocrine responses influence the brain function and affect mental health [3].

The interactions between gene and environment are of special interest for the development of neurogenesis and neurobiochemical systems that work into the Hypothalamic-Pituitary-Adrenal (HPA) axis and how they can result in emotional responses [1, 2]. The environment is a strong activator of the HPA axis, the complex feedback-regulated neuroendocrine system controlling cortisol secretion and physiological stress responses in mammals [1, 4]. Some data highlights the cumulative burden of social stress and HPA axis activation facilitating the onset of mental disorders [1, 5]. Severe and chronic stress may clearly lead to changes in the prefrontal and in limbic systems with lifelong adjustments in region-specific gene expression, neural plasticity, neuroendocrine function and behavioural response to stressors [4, 5].

One of the established impact on mental health is of the modern city life [1, 6]. Almost all urban subjects tend to be healthier than rural population, as they have superior educational, economic and healthcare conveniences that large cities may offer [6]. But interestingly, the opposite seems to be true for mental health—psychiatric disorders are more frequent in urban areas [1, 7]. Evidence suggests a dose-dependent relationship between psychosis onset, severity and its prognosis when the subject is exposed to urban environments [8].

The urban specific features of demand and adaptation are highly complex and have an enormous variability, and they are useful as an agent for a set of environmental influences in the city. Carta *et al*. [9] presented us with some interesting hypotheses about how cities’ noise and light pollution might influence our mental health and increase the risk for bipolar disorder. They [9] suggested that we may be facing an evolutionary decoupling of habits and adaptive demands. From an evolutionary perspective, the authors [9] assumed that having an excess of energy during a sleepless night episode may have had an adaptive effect. They pointed out that we were accustomed to resting at night and awakening due to light (or noise) may be undoubtedly associated with a stress reaction that urges a consumption of mental and physical resources [9]. It can be supposed and scientifically tested that if the city demands that biological rhythms be broken, people with a basic predisposition to living with biologic rhythms different from what was considered normal in a previous moment of human life may be in an adaptive state [9].

For their point of view [9], people who are driven to novelty-seeking may be, for example, explorers with hyper-rhythmic temperaments, which are also consistent with studies on migrants by will and not as refugees, as observed in studies conducted by Carta *et al*. [10]. The psychopathological changes and demands nowadays in big cities would be the disordered side of those who find it difficult to adapt to the demands of non-stop activities. But the new lifestyle might also influence those who adapt and show behaviors, reactions and responses that might resemble bipolar disorder, but may be just an adaptative behavior [9]. This may also justify some overdiagnosis of bipolar disorder II in some psychiatric centers. Supporting their hypotheses and a general worry of many psychiatrists, some surveys have found that mood disorders and bipolar disorder continue to increase in Western societies [11]. For Carta *et al*. [9], a possible explanation for this paradox may be that those who have so-called “predisposition to mood disorders” but do not become ill will live better and reproduce in the new world of constant activity.

Sleep patterns are increasingly jeopardized by recent changes in the challenging human modern life [9]. Individuals with poor quality of sleep might be more vulnerable to the impact of road traffic noise on mental health even if it is difficult to understand the precise mechanism. Carta *et al*. [9] directed our attention to the idea that it is still undetermined whether those affected by road traffic noise were already poor sleepers or poor sleep was the first manifestation of impairment due to road traffic, thus being the first step in mental disorder onset [9].

Artificial light changes daily rhythms by allowing the presence, during hours of natural darkness, of activities normally performed during daylight hours, such as food intake or social meetings [9]. This has a great impact on the immune-endocrine circadian timing mechanisms and the other endogenous rhythms that have evolved to ensure that human behavior are more efficient when synchronized with variations in light and with other environmental circumstances such as weather and seasons [9, 12]. Some studies suggested that sleep-wake cycle interruptions and artificial light pollution might be triggering features for bipolar disorder [13].

Although melatonin steroid-induced mechanisms are very complex, in general, they decrease estradiol and increase progesterone levels [14], so the block of melatonin at night, due to light pollution, unbalances the estradiol / progesterone ratio in favor of estradiol which may be relevant in bipolar disorder [1, 9]. Carta *et al*. [9] advanced the hypothesis that the blockade of night-time production of melatonin due to light pollution may play an important role in the genesis of bipolar disorder also as a consequence of the effect that melatonin exerts on the stability of steroid hormones.

Another challenge for future research tests will be the development of animal models that could be compared to the complexity of environmental challenges in the modern world [1]. And we must always consider that modern life and its demanding are changing every day, mainly increasing its stress factors. A long-term study of risk and resilience relationship to mental disorders onset could also present us the perspective of preventive measures to psychiatric disorders and promote a better life for people daily challenged by the rapid modern world demands.
